# Translation Initiation Machinery as a Tumor Selective Target for Radiosensitization

**DOI:** 10.3390/ijms221910664

**Published:** 2021-10-01

**Authors:** Stacey L. Lehman, Evan D. Wilson, Kevin Camphausen, Philip J. Tofilon

**Affiliations:** Radiation Oncology Branch, National Cancer Institute, Bethesda, MD 20892, USA; stacey.lehman@nih.gov (S.L.L.); evan.wilson@nih.gov (E.D.W.); camphauk@mail.nih.gov (K.C.)

**Keywords:** radiosensitization, eIF4E, eIF4G, eIF4A, mTOR, ribosome biogenesis

## Abstract

Towards improving the efficacy of radiotherapy, one approach is to target the molecules and processes mediating cellular radioresponse. Along these lines, translational control of gene expression has been established as a fundamental component of cellular radioresponse, which suggests that the molecules participating in this process (i.e., the translational machinery) can serve as determinants of radiosensitivity. Moreover, the proteins comprising the translational machinery are often overexpressed in tumor cells suggesting the potential for tumor specific radiosensitization. Studies to date have shown that inhibiting proteins involved in translation initiation, the rate-limiting step in translation, specifically the three members of the eIF4F cap binding complex eIF4E, eIF4G, and eIF4A as well as the cap binding regulatory kinases mTOR and Mnk1/2, results in the radiosensitization of tumor cells. Because ribosomes are required for translation initiation, inhibiting ribosome biogenesis also appears to be a strategy for radiosensitization. In general, the radiosensitization induced by targeting the translation initiation machinery involves inhibition of DNA repair, which appears to be the consequence of a reduced expression of proteins critical to radioresponse. The availability of clinically relevant inhibitors of this component of the translational machinery suggests opportunities to extend this approach to radiosensitization to patient care.

## 1. Introduction

Radiation therapy, along with surgery and chemotherapy, is a major treatment modality for cancer. Almost two-thirds of patients with solid tumors receive radiation therapy as part of their treatment regimen [1]. Radiation is used to treat numerous cancer types, including glioblastoma, prostate cancer, lung cancer, melanoma, and head and neck cancer. The clinically relevant form of radiation used in cancer therapy is ionizing radiation, which is most often delivered as high energy photons by a linear accelerator [2]. Although radiation therapy is tailored to each patient, a typical treatment course lasts for 6 to 8 weeks with 5 to 6 daily treatments per week; the dose for each treatment fraction is normally 1.8 to 2 Gy [2]. The dose of radiation a patient can receive is often limited by the toxicity to nearby normal tissue. Given the widespread use of radiation therapy, the development of drugs that can selectively sensitize tumor cells to radiation will have immense clinical benefits.

The development of molecularly targeted radiosensitizing agents depends on a thorough understanding of the fundamental processes that regulate cellular radioresponse. Towards this end, analyses of the post-translational modification of existing proteins have led to critical insights into the events and signaling pathways mediating essential components of radioresponse (e.g., DNA repair, cell cycle checkpoint activation, apoptosis) and has provided a source of targets for radiosensitization. Radiation has also been shown to selectively regulate mRNA translation, a process that operates independently from changes in transcription [3,4]. Characteristics of radiation-induced translational control of gene expression include commonly affected mRNAs among cell lines generated from the same tissue type [5], which is in contrast to radiation-induced changes in gene transcription [6]. In addition, and critical to interpreting its function consequences, there is a correlation between the mRNAs whose translational activity is affected by radiation and expression of their corresponding protein [1,2], again in contrast to radiation-induced changes in gene transcription [7]. Thus, data generated to date indicate that translational control is a fundamental component of cellular radioresponse, which implies that molecules participating in this process can serve as determinants of radiosensitivity.

A defining characteristic of tumor cells is the reprograming of translational control to selectively enhance the translation of genes mediating cell proliferation, survival and other processes critical to the neoplastic phenotype [8] with a number of the proteins mediating mRNA translation often overexpressed in human tumors (see below). This difference between tumor and normal cells suggests that the translational machinery may also provide tumor specific targets for radiosensitization. There are a number of post-transcriptional processes participating in gene expression, such as splicing, mRNA maturation (e.g., capping, polyadenylation) and nuclear export. However, the final event is translation, which occurs in 3 steps: initiation, elongation and termination. Initiation is the rate-limiting step in translation; results generated to date indicate that translational control operates primarily at the initiation step [9]. In this review, we summarize results implicating the translation initiation machinery as a determinant of radiosensitivity and the potential of its individual components to provide clinically relevant targets for radiosensitization. Whether processes downstream from initiation (i.e., elongation and termination) affect radiosensitivity have not been reported.

## 2. Translation Initiation: eIF4F

**Figure 1:**
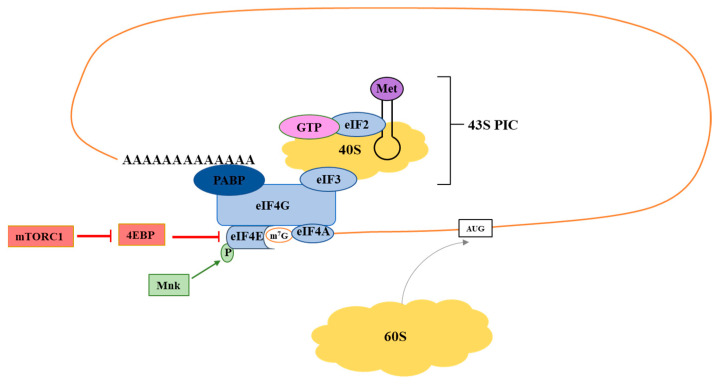
Overview of cap-dependent translational initiation. The eIF4F complex consists of eIF4G, eIF4E, and eIF4A. eIF4E is the m^7^G mRNA cap binding protein, eIF4A is an RNA helicase, and eIF4G is a scaffolding protein. To initiate translation, eIF4F associates with the mRNA cap to recruit the 43S preinitiation complex (PIC). The Mnk kinases activate eIF4F through phosphorylation of eIF4E. mTORC1 activates eIF4F by phosphorylation of 4E-BP, which prevents its inhibitory binding to eIF4E.

The initiation step of translation involves the assembly eIF4E, eIF4G and eIF4A into the eIF4F initiation complex. In eukaryotic cells, the majority of translation occurs in a cap-dependent manner with eIF4F binding to the 7-methyl guanosine (m^7^G) cap on the 5′ end of an mRNA, which subsequently recruits the 43S ribosome preinitiation complex to the transcript (Figure 1) [9]. eIF4E plays a critical role as the cap binding protein of eIF4F; it also binds to eIF4G to promote assembly of the eIF4F complex [10]. eIF4G is a scaffolding protein that recruits eIF4A and other initiation factors and regulatory molecules [11]. eIF4A functions as a helicase that unwinds complex secondary structures in mRNAs to facilitate ribosome scanning [12]. Formation of eIF4F is the rate-limiting step in translation initiation; each component of the eIF4F complex has been implicated as a determinant of tumor cell radiosensitivity.

### 2.1. eIF4E

eIF4F formation is highly dependent on the availability of eIF4E. The level of eIF4E is regulated through transcription as well as through interactions with the 4E-BP family of binding proteins; its activity is also affected by serine phosphorylation [13]. The multiple processes regulating the availability of eIF4E allow for the rapid modification of gene translation in response to various forms of stress and oncogenic signaling. However, the reliance on eIF4E for translation varies among genes. Elevated levels of eIF4E preferentially enhance the translation of mRNAs with long, highly structured 5′ untranslated regions (UTRs), which tend to encode proteins related to cell proliferation and survival. In addition, eIF4E selectively enhances the nuclear export of mRNAs, many of which encode proteins involved in oncogenesis [10]. Consistent with such molecular observations, increased eIF4E levels have been associated with malignant progression and poor therapeutic outcome [14,15,16], and in preclinical models, targeting eIF4E has been reported to inhibit tumor growth [13].

Given its function in translational control and its contribution to the neoplastic phenotype, the role of eIF4E as a determinant of radiosensitivity has also been investigated. Knockdown of eIF4E using siRNA resulted in an increase in the radiosensitivity of three tumor cell lines yet had no effect on the radiosensitivity of the two normal cell lines [17]. These results suggested that eIF4E contributes to survival after irradiation of tumor, but not normal, cells. The radiosensitization of the tumor cells could not be attributed to cell cycle redistribution into a radiosensitive phase or to an enhancement in apoptosis. MDA-MB-231 breast adenocarcinoma cells treated with siRNA against eIF4E had essentially the same cell cycle distribution as cells treated with a nontargeting siRNA 72 h post-transfection, and radiation alone did not result in significant apoptosis in cells transfected with non-targeting siRNA or siRNA against eIF4E [17]. However, eIF4E knockdown did increase the frequency of radiation-induced mitotic catastrophe and delayed the dispersion of radiation-induced γH2AX foci, indicating that the radiosensitization is ultimately the result of an inhibition of radiation-induced DNA DSB repair. Linking eIF4E to radiation-induced translational control was a RIP-chip analysis showing that radiation increased eIF4E binding to >1000 unique transcripts. Importantly, a significant number of the genes whose eIF4E binding was modified by radiation participate in aspects of the DNA damage response including double strand break repair and cell cycle checkpoints. This indicates that eIF4E may influence DNA repair both directly through expression of repair proteins and indirectly through expression of proteins involved in signaling pathways critical to radioresponse. Although additional experiments beyond mRNA binding are necessary to determine the role of eIF4E in regulating the translation of these transcripts, this initial study suggests that eIF4E participates in the radiation-induced translational control of gene expression and to be a tumor-selective target for radiosensitization.

Targeting eIF4E as a cancer treatment strategy has primarily focused on antisense oligonucleotides (ASO) (Table 1). Preclinical studies using ASOs have shown reductions in eIF4E levels accompanied by slowing of tumor proliferation in vitro and in vivo [18,19,20]. A clinical trial of the ASO LY2275796 in patients with solid tumors, while failing to observe a tumor response, found tumor eIF4E levels to be generally reduced at the protein and mRNA levels [21]. Although, as suggested by the authors, these results support the study of this eIF4E ASO in combined with other cancer treatment modalities, the effect of a combination with radiotherapy (to our knowledge) have not been reported.

Because eIF4E is not an enzyme, pharmacological approaches to its inhibition have been primarily limited to ribavirin, a guanosine nucleoside analogue. Initially identified as an anti-viral agent, ribavirin was shown to bind to eIF4E and to reduce its binding to capped RNA [23,24]. Although there is some controversy as to the specific mechanism, ribavirin has been reported to reduce the levels of eIF4E dependent proteins and in preclinical studies to inhibit growth of various tumor types [23,25]. Clinical trials (Table 1) of ribavirin have been completed or are ongoing against multiple tumor types and in combination with a variety of chemotherapeutic or targeted agents [26]. Consistent with its inhibition of eIF4E activity, ribavirin has also been shown to enhance the in vitro radiosensitivity of breast [17] and glioma cells [27]. Huq et al. reported that ribavirin enhances the radiosensitivity of nasopharyngeal carcinoma cells in vitro and when grown as flank tumor xenografts [28]. These results suggest that ribavirin provides a clinically relevant approach to inhibiting eIF4E and inducing tumor radiosensitization.

### 2.2. eIF4G

eIF4G functions as a scaffolding protein to promote the assembly of the eIF4F complex and serves as a docking station for other proteins that regulate translation, such as eIF3 and poly(A)-binding protein (PABP) (Figure 1) [11]. Thus, it also plays a crucial role in the recruitment of ribosomes to mRNAs. Its overexpression has been documented in various cancer types, including breast cancer and squamous cell lung cancer [29,30,31].

The role of eIF4G in cellular radioresponse has been investigated in breast cancer [32]. Knockdown of eIF4G with shRNA was shown to radiosensitize four breast cancer cell lines. At the molecular level, eIF4G knockdown both delayed and prolonged the formation of radiation-induced γH2AX foci. γH2AX foci accumulated in control cells within minutes of radiation and resolved by 24 h post-irradiation, while eIF4G knockdown cells did not show appreciable accumulation of γH2AX until one hour post-irradiation, with the foci persisting at 24 h after irradiation. Analysis of cell cycle distribution also showed that a smaller percentage of eIF4G knockdown cells accumulated in G_2_/M after radiation. Polysome profiling was utilized to measure translational changes in gene expression in irradiated cells with and without silenced eIF4G. This technique separates mRNAs based on density in sucrose gradients: efficiently translated mRNAs have many ribosomes bound (polysomes) and, therefore, sediment lower in a gradient than poorly translated mRNAs with few or no associated ribosomes [33]. These experiments demonstrated that eIF4G regulated the translation of a number of genes involved in the DNA damage response, cell cycle regulation, and survival, including ATM, 53BP1, GADD45A, and XIAP. The targets of eIF4G identified in this study suggest that eIF4G directly promotes the expression pro-survival signaling proteins and proteins directly involved in DNA repair. Therefore, eIF4G-mediated translational control likely explains the function of this protein in cellular radioresponse as opposed to a direct effect of eIF4G at sites of DNA damage.

eIF4G has been pharmacologically targeted through small molecules that disrupt the eIF4G-eIF4E interaction. One such molecule is 4EGI-1 [34]. This drug has been shown to inhibit the translation of transcripts with highly structured 5′ UTRs, such as myc, Bcl-XL, cyclin D, and survivin [35]. Treatment with 4EGI-1 results in growth inhibition and apoptosis induction in several cancer cell lines [13]. Although this pharmacological approach has not been extensively studied in combination with radiation, Wang et al. have published initial results indicating that 4EGI-1 radiosensitizes nasopharyngeal carcinoma (NPC) cells [36]. Treatment with 4EGI-1 24 h before radiation significantly radiosensitized the HNE1 NPC cell line. Cells treated with the combination of 4EGI-1 and radiation expressed higher levels of cleaved PARP and death receptor 5 as compared to cells treated with radiation alone, indicating that 4EGI-1 enhanced apoptosis in irradiated cells. While small molecules targeting eIF4G have not yet made it to the clinic, this represents a possible avenue for future studies.

### 2.3. eIF4A

The third member of eIF4F is the RNA helicase eIF4A. It unwinds mRNA secondary structure, which promotes ribosomal scanning through complex 5′ UTRs. Like eIF4E and eIF4G, eIF4A overexpression has been documented in a variety of cancer types, including breast, lung, and cervical carcinoma [31,37,38]. eIF4A overexpression has been shown to accelerate cancer development in mouse models of leukemia [39], and, conversely, its inhibition has been shown to slow tumor growth [12].

eIF4A is the only enzymatic member of eIF4F, and, consequently, several inhibitors targeting its helicase function have been identified and developed. Hippuristanol and pateamine A are two natural compounds with eIF4A inhibitory activity. Hippuristanol is an allosteric inhibitor that stabilizes the closed confirmation of eIF4A, while pateamine A irreversibly induces non-specific binding of eIF4A to RNA, depleting eIF4A from eIF4F [40,41]. A family of related inhibitors known as rocaglates includes many synthetic and natural products, such as silvestrol, Roc A, CR-1-31-B, and SDS-1-021. These compounds also generally function by inducing non-specific binding of eIF4A to RNA, but unlike pateamine A, the binding is reversible [42]. Many studies have documented that eIF4A inhibitors can inhibit tumor growth both in vitro and in vivo [12]. Furthermore, the rocaglate-like eIF4A inhibitor zotatifin (eFT226) has recently entered a phase I/II clinical trial for solid tumors (Table 1) [43,44].

Despite the pharmacological agents available to target eIF4A as compared to the other components of eIF4F, there is limited information regarding the role of this RNA helicase in radiosensitivity. Silvestrol has been shown to sensitize mouse models of lymphoma to doxorubicin, which suggests that eIF4A plays a role in the response to DNA-damaging agents [42]. Initial studies with silvestrol [45] and eIF4A knockdown [46] provided the first evidence that eIF4A inhibition can radiosensitize tumor cells. Treatment of T-47D ductal breast carcinoma cells with 1 nM silvestrol significantly reduced clonogenic survival of irradiated cells. Silencing eIF4A1 with shRNA in HeLa and SiHa cells also significantly decreased clonogenic survival as compared to parental cells after irradiation. eIF4A1 knockdown cells also displayed prolonged γH2AX phosphorylation after radiation, as detected by western blot. The entry of eIF4A inhibitors into clinical trials supports further research into their effects on tumor cell radiosensitivity.

## 3. Signaling Molecules Regulating eIF4F Activity

### 3.1. mTOR

**Figure 2:**
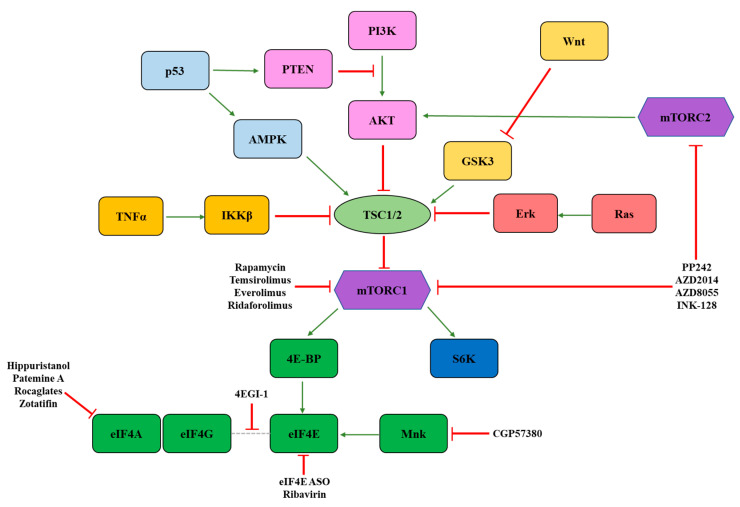
Overview of mTORC signaling and pathway inhibitors. mTORC1 is a funnel factor that receives inputs from numerous signaling pathways, including PI3K, Wnt, Ras, p53, and TNFα. mTORC1 transmits these signals to eIF4F through phosphorylation of 4E-BPs. Pathway inhibitors discussed in the text are indicated.

Upstream signaling molecules can regulate eIF4F activity to coordinate global translation and the translation of specific transcripts in response to stress and bioenergetic demands. One of the most studied eIF4F regulators is mechanistic target of rapamycin (mTOR) (Figure 2). mTOR is a member of two distinct protein complexes: mTORC1 and mTORC2. In addition to mTOR, mTORC1 and mTORC2 both contain the proteins Deptor and mLST8. Unique to mTORC1 are Raptor and PRAS40, while Rictor, mSin1, and Protor are specific to mTORC2 [47]. mTORC1 directly influences translation through phosphorylation of S6K1 and 4E-BP. Relevant to eIF4F activity, phosphorylated 4E-BP is unable to bind and sequester eIF4E. Thus, activation of mTORC1 stimulates eIF4F activity by increasing the amount of eIF4E available to form a translationally active complex [48]. The mTORC2 signaling pathway is less understood, and, to date, has no known role in directly regulating eIF4F. However, mTORC2 phosphorylates and activates AKT, which is an upstream activator of mTORC1 [49].

mTORC1 functions as a “funnel factor” [50] at which the inputs of many different signaling pathways converge to impinge upon the translational machinery (Figure 2). For example, growth factor receptors activate mTORC1 through the PI3K-AKT signaling pathway, which inactivates the mTOR inhibitory complex TSC1/2 [51]. Ras signaling and TNFα signaling also stimulate mTORC1 activity through Erk-mediated inactivation of TSC1/2 and IKKβ-mediated inactivation of TSC1/2, respectively [52,53]. Activation of the Wnt pathway inhibits GSK3, which is a TSC1/2 activator [54]. In contrast, activation of p53 signaling inhibits mTORC1 activity by stimulating TSC1/2 directly or indirectly through AMPK. p53 can also activate PTEN, which inhibits the AKT pathway and therefore inhibits mTORC1 [55].

mTOR’s central role in relaying information from numerous signaling pathways to eIF4F has made it an attractive target for cancer therapy and for radiosensitization. Initial studies into mTOR as a target for tumor radiosensitization utilized rapamycin and its derivatives, which are allosteric mTOR inhibitors. These experiments (reviewed in [56] and [57]) produced mixed results. For example, Holler et al. [58] found that rapamycin sensitized H661 and H460 cells but not SK-MES-1, HTB-182 or MDA-MB-231. Eshleman et al. [59] reported that rapamycin did not radiosensitize U87 cells grown in monolayers, but it did radiosensitize the same cells grown as spheres or as xenografts. In contrast, Weppler et al. [60] reported no radiosensitization of U87 xenografts with rapamycin. These differences in results between cell lines and treatment protocols suggest that cell line mutational background, tissue of origin, and/or the dose and time of drug and radiation can all influence the effectiveness of rapamycin as radiosensitizer.

However, rapamycin and its analogs are incomplete inhibitors of mTOR, which further complicates the understanding of mTOR’s role in cellular radioresponse. Rapamycin only partially inhibits mTORC1 and does not inhibit mTORC2 [61]. With respect to mTORC1, rapamycin inhibits its phosphorylation of S6 kinase 1, but it is not an effective inhibitor of mTORC1′s phosphorylation of 4E-BP [62]. The second-generation ATP competitive mTOR inhibitors circumvent these issues by inhibiting both mTORC1 and mTORC2 and by more completely inhibiting mTORC1-mediated 4E-BP phosphorylation [61]. A direct comparison of rapamycin and the ATP competitive inhibitor PP242 in the U251 glioblastoma cell line and MDA-MB-231 breast adenocarcinoma cell line demonstrated radiosensitization by PP242 but not rapamycin [63]. Similar results were obtained in the SUM149 inflammatory breast carcinoma cell line with the comparison of PP242 and the rapalog RAD001 [64]. In both studies, PP242 radiosensitized tumor cells both in vitro and in vivo and inhibited the repair of radiation-induced DNA damage. Importantly, PP242 had no effect on the radiosensitivity of a normal human fibroblast cell line [63].

Additional studies with other ATP competitive mTOR inhibitors support mTORC1/2 as a target for tumor cell radiosensitization. AZD2014 was shown to radiosensitize glioblastoma stem-like cells (GSCs) in vitro when added one hour before irradiation [65]. Like PP242, it delayed the repair of radiation-induced DNA damage. In vivo, AZD2014 radiosensitized the GBMJ1 GSC line grown orthotopically in mice [65]. AZD2014 also enhanced radiation-induced tumor cell killing in the oral squamous cell carcinoma (OSCC) cell lines SCC4 and SCC25 and in primary OSCC cells directly obtained from patients [66]. INK-128 (TAK-228 in the clinic, see Table 1) induced radiosensitization in PSN1, PANC-1, and MiaPaCa-2 pancreatic carcinoma cell lines in vitro and in PSN1 grown subcutaneously in vivo [67]. As for PP242, INK128 did not affect the radiosensitivity of normal cells [67]. In diffuse intrinsic pontine glioma cell lines, INK128 in combination with radiation was shown to decrease proliferation, as measured by BrdU incorporation, and increase apoptosis, as measured by cleaved caspase 3, as compared to either treatment alone [68]. Polysome profiling studies with PP242 [64] and INK128 [67] in irradiated cells showed a reduction in the translation of genes involved the DNA damage response, which provides a mechanistic link between mTOR’s role in translation and cellular radioresponse. This is consistent with the results obtained with eIF4E RIP-chip [17] and eIF4G knockdown [32] studies, suggesting that the cap binding complex and its regulation play an important role in radiation-induced translational regulation of gene expression. Altogether, the gene expression studies discussed here indicate that eIF4F promotes the survival of irradiated tumor cells through enhanced translation of mRNAs involved in the DNA damage response, DNA repair, cell cycle checkpoints, and inhibition of cell death. Future studies addressing cell line and dose-dependent effects will be helpful to parse out their influence on radiation-induced translational control.

To date, four drugs that inhibit mTORC1 and two that inhibit mTORC1/2 have been used in the clinic (Table 1). Sirolimus (rapamycin), the first in class mTORC1 inhibitor, was FDA approved in 1999 for the prevention of organ transplant rejection and in 2015 for the treatment of lymphangioleiomyomatosis (www.fda.gov, accessed on 24 August 2021). Numerous trials of Sirolimus in combination with chemotherapy have been finished but no additional benefit over the standard of care has been noted (www.clinicaltrials.gov, accessed on 24 August 2021). Similarly, there have been several trials using Sirolimus in combination with radiotherapy, but no positive outcomes have been reported. The second drug Temsirolimus, a rapamycin prodrug, was FDA approved in 2007 for the treatment of renal cell carcinoma (www.fda.gov, accessed on 24 August 2021). Two Phase 1 studies of Temsirolimus plus radiotherapy showed the combination to be safe [69,70], and ongoing studies will determine if there is a synergistic effect from the combination (www.clinicaltrials.gov, accessed on 24 August 2021). Everolimus, a derivative of Sirolimus, was FDA approved (2009) for the prevention of organ transplant rejection and for the treatment of multiple tumor types (renal cell, giant cell astrocytoma, and pancreatic neuroendocrine tumors) (www.fda.gov, accessed on 24 August 2021). Three studies have been published using a combination of Everolimus and radiotherapy, with the two Phase 1 studies showing a safe side effect profile [71,72] and the one completed Phase 2 trial showing no benefit to the addition of Everolimus to temozolomide and radiation in patients with glioblastoma [22]. The last mTORC1 specific drug, Ridaforolimus, had a positive Phase 3 trial in soft tissue sarcoma, but no studies are ongoing in combination with radiotherapy (www.clinicaltirals.gov, accessed on 24 August 2021). Two drugs that inhibit both mTORC1 and 2 are also in clinical development (AZD8055 and Sapaniserib (TAK228/INK-128)). There are two Phase 1 studies that have been published with AZD8055, both of which show a safe profile [73,74]; however, there are no ongoing trials of AZD8055 in combination with radiotherapy. Likewise, there are safety trials for Sapanisertib [75,76] but no ongoing studies in combination with radiotherapy. Thus far, the four drugs that target only mTORC1 have not shown an enhancement of radiation sensitivity in the completed clinical trials, and the two drugs that inhibit both mTORC1 and 2 are much earlier in their clinical development.

### 3.2. Mnk

The MAP kinase interacting kinases 1 and 2 (Mnk 1 and 2) regulate eIF4E through phosphorylation of S209. This phosphorylation event has been shown to promote the translation and nuclear export of genes involved in proliferation and survival. Thus, Mnk activity is important for the oncogenic functions of eIF4E [77,78]. Both Mnk1/2 double knockout mice and eIF4E^S209A^ mutant mice are viable and fertile, indicating that Mnk activity is dispensable for normal growth and development [78,79]. Targeting Mnk is therefore an additional approach for cancer therapy. As with many of the proteins involved in translation initiation, Mnks are overexpressed in several different cancer types, which is often associated with poor prognosis [80].

In the context of radiotherapy, the Mnk inhibitor CGP57380 has been shown to radiosensitize nasopharyngeal carcinoma cells in vitro [81]. CNE1 cells pre-treated with 25 µM CGP57380 for 24h prior to radiation exhibited decreased clonogenic survival as compared to untreated cells. Sulforhodamine B assays also demonstrated that radiation and CGP57380 synergistically inhibited cell growth. CNE1 and HNE1 cells treated with radiation and CGP57380 showed increased Annexin V staining by flow cytometry and increased cleaved PARP by Western blot as compared to either treatment alone, suggesting that apoptosis is increased with the combination treatment. In pancreatic carcinoma, Mnk-mediated phosphorylation of eIF4E was responsible for translational upregulation of Sox2, which promoted tumor recurrence after radiation [82]. In a co-culture model of tumor recurrence after radiation, SW1990 and BxPC-3 cells treated with 10Gy were shown to stimulate the proliferation of non-irradiated cells from the same cell line. Treatment with CGP57380 significantly reduced the ability of irradiated cells to stimulate the growth of their non-irradiated counterparts, suggesting that Mnks may play a role in tumor cell repopulation after radiation. Over 40 Mnk inhibitors in addition to CGP57380 have been developed to date, with three of them (BAY1143269, Tomivosertib (eFT508) and ETC-1907206) having completed early Phase1/2 studies. However, there are no ongoing studies of a MNK inhibitor in combination with radiotherapy (Table 1) [80].

## 4. Ribosome Biogenesis

Critical to translation initiation is the availability of ribosomes. Thus, as an alternative to components of eIF4F, translation initiation can also be targeted by inhibiting ribosome biogenesis. Ribosome biogenesis is a complex process consisting of the production and modification of ribosomal RNA and proteins into mature, translationally active ribosome subunits. The majority of ribosome biogenesis takes place in the nucleolus, where RNA polymerase I (Pol I) transcribes rDNA into the 47S rRNA precursor. 47S rRNA undergoes cleavage and nucleotide modification to produce mature 18S, 5.8S, and 28S rRNA [83]. Ribosomal proteins are synthesized in the cytoplasm and are transported into the nucleus where they assemble with processed rRNA into pre-40S and pre-60S ribosomal subunits. These subunits are then exported into the cytoplasm where they undergo further modification into mature ribosomes [84]. Because ribosome biogenesis is closely coupled to cellular growth and proliferation rates, it is frequently upregulated in cancer. Cancer cells are nearly always characterized by abnormal nucleolar size and morphology and frequently exhibit increased Pol I expression and activity [85]. Thus, ribosome biogenesis has been suggested as a tumor selective target for cancer therapy.

The most direct approach to inhibiting ribosome biogenesis is through the inhibition of Pol I, which exclusively transcribes rDNA into rRNA. A number of chemotherapeutic agents, such as actinomycin D, cisplatin, doxorubicin, mitomycin C, etoposide, and 5-fluorouricil, nonspecifically inhibit Pol I through various mechanisms [86]. For example, platinum based alkylating agents like cisplatin form platinum adducts throughout the genome, which can impede Pol I transcription and sequester other members of the Pol I transcription machinery [87]. Actinomycin D, on the other hand, preferentially intercalates in the genome at GC-rich sites, which causes polymerase stalling. Due to the GC-rich nature of rDNA, Pol I transcription is especially sensitive to actinomycin D [87]. Specific inhibitors of Pol I have also been developed, including Quarfloxin (CX-3543), Pidnarulex (CX-5461), and BMH-21. Quarfloxin binds to G-quadruplex structures in rDNA, which causes nucleolin dissociation and inhibition of Pol I elongation [88]. Pidnarulex inhibits the formation of the Pol I pre-initiation complex [89], while BMH-21 is an intercalating agent that causes disassembly of the Pol I complex [90]. Both Quarfloxin and Pidnarulux have completed phase I clinical trials in cancer patients (Table 1), while BMH-21 is under pre-clinical development [87]. Although these inhibitors have been used in combination with other chemotherapy (www.clincaltrials.gov, accessed on 24 August 2021), it remains to be seen if Pol I inhibition enhances tumor radiosensitivity.

The initial suggestion that ribosome biogenesis may provide a target for radiosensitization came from work with the XPO1 inhibitor Selinexor. XPO1 is a critical receptor mediating the nuclear-cytoplasmic export of pre-ribosomal subunits. In complex with RanGTP in the nucleus, it associates with pre-40S and pre-60S subunits through adaptor proteins that contain nuclear export signals (NES) to drive their transport to the cytoplasm [91]. Selinexor covalently binds to the NES binding pocket on XPO1, which inhibits its ability to interact with cargo molecules [92]. Treatment of glioblastoma cell lines with Selinexor resulted in the cytoplasmic loss and nuclear accumulation of 18S and 5S rRNA, which correlated with inhibition of translation and protein synthesis. These effects were accompanied by an inhibition of DNA double strand break repair and the radiosensitization of tumor cells both in vitro and in vivo. The inhibition of ribosome export, reduction in protein synthesis and radiosensitization were not observed in normal cells, indicating that Selinexor functions in a tumor specific manner [93].

Selinexor was approved by the FDA in 2019 for the treatment of relapsed refractory multiple myeloma (www.fda.gov, accessed on 24 August 2021). Since then, numerous trials of combination chemotherapy in a multitude of disease histologies have been undertaken. Additionally, there are three ongoing studies of the combination of Selinexor with radiotherapy. The first is a study being conducted at the National Institutes of Health (USA) of the combination of Selinexor, Temodar and radiotherapy for patients with glioblastoma. Importantly, this study is only using Selinexor in combination with the radiation portion of the treatment to determine if the drug is a radiation sensitizer. Two additional studies are also ongoing: one in primary and recurrent GBM and one in primary rectal cancer (www.clinicaltrials.gov, accessed on 24 August 2021).

## 5. Conclusions

Translation initiation, which is the rate-limiting step in translation and exerts a major influence on the translational control of gene expression, differs significantly between tumor and normal cells. The proteins involved are often over expressed in tumors and the process of translation initiation is critical to the tumor cell required expression of survival, proliferative and oncogenic proteins. Accordingly, translation initiation is considered a target for cancer treatment. An analogous situation appears to exist for radiosensitivity. Radiation-induced translational control of gene expression differs between tumor and normal cells [5] suggesting that translation initiation may differentially influence the radiosensitivity of tumor and normal cells. Indeed, studies to date targeting the molecular machinery of translation initiation including ribosome biogenesis suggest that the radioresponse of tumor cells is more dependent on gene translation than that of normal cells. Whereas many molecular determinants of radiosensitivity have been identified, such as DNA repair proteins and those involved in cell cycle checkpoint activation, the development of clinically relevant radiosensitizers requires the identification of proteins that preferentially influence the radioresponse of tumor cells. The molecules mediating translation initiation appear to meet the requirement for tumor specificity. As inhibitors of translation initiation move through clinical trials as single agents, they will also be candidates for evaluation in combination with radiotherapy.

## Figures and Tables

**Table 1:** Translation initiation inhibitors in clinical trials. Summary of the usage of inhibitors discussed this article that are in clinical trials or have FDA approval.

Agent	Target	Clinical Usage	Radiotherapy Trial
LY2275796 (ASO)	eIF4E	Phase 1 complete; not as a sensitizer	no
Ribavirin	eIF4E	FDA approved; not as a sensitizer	no
Zotatifin (eFT226)	eIF4A	Phase 1	no
Sirolimus (rapamycin)	mTORC1	FDA approved; not as a sensitizer	no
Temsirolimus (CCI-779)	mTORC1	FDA approved; not as a sensitizer	yes-ongoing
Everolimus (RAD-001)	mTORC1	Phase II; as an RT sensitizer	yes-no benefit [22]
Ridaforolimus (AP23573)	mTORC1	Phase III; drug only combinations	no
AZD8055	mTORC1/2	Phase 1	no
Sapanisertib (Tak-228)	mTORC1/2	Phase2/3; not as a sensitizer	no
BAY1143269	MNK1	Phase 1; not as a sensitizer	no
Tomivosertib (eFT508)	MNK1/2	Phase 1/2; not as a sensitizer	no
ETC-1907206	MNK1/2	Phase 1; not as a sensitizer	no
Quarfloxin (CX-3543)	RNA pol I	Phase 1; not as a sensitizer	no
Pidnarulex (CX-5461)	RNA pol I	Phase 1; not as a sensitizer	no
Selinexor (KPT-330)	Ribosome Biogenesis	FDA approved; not as a sensitizer	yes-ongoing

## Data Availability

Not applicable.
